# The low-symmetry lanthanum(III) oxotellurate(IV), La_10_Te_12_O_39_

**DOI:** 10.1107/S1600536813012191

**Published:** 2013-05-18

**Authors:** Peng Li Wang, Yurij Mozharivskyj

**Affiliations:** a Department of Chemistry and Chemical Biology, McMaster University, 1280 Main Street West, Hamilton, Ontario, L8S 4M1, Canada

## Abstract

Single crystals of deca­lanthanum(III) dodeca­oxotellurate(IV), La_10_Te_12_O_39_, were obtained by reacting La_2_O_3_ and TeO_2_ in a CsCl flux. Its crystal structure can be viewed as a three-dimensional network of corner- and edge-sharing LaO_8_ polyhedra with Te^IV^ atoms filling the inter­stitial sites. The Te^IV^ atoms with their 5*s* ^2^ electron lone pairs distort the LaO_8_ polyhedra through variable Te—O bonds. Among the six unique Te sites, four of them define empty channels extending parallel to the *a* axis. The formation of these channels is a result of the stereochemically active electron lone pairs on the Te^IV^ atoms. The atomic arrangement of the Te—O units can be understood on the basis of the valence shell electron pair repulsion (VSEPR) model. A certain degree of disorder is observed in the crystal structure. As a result, one of the five different La sites is split into two positions with an occupancy ratio of 0.875 (2):0.125 (2). Also, one of the oxygen sites is split into two positions in a 0.559 (13):0.441 (13) ratio, and one O site is half-occupied. Such disorder was observed in all measured La_10_Te_12_O_39_ crystals.

## Related literature

For the structures of related rare-earth oxotellurates(IV), see: Castro *et al.* (1990 ▶); Weber *et al.* (2001 ▶); Meier *et al.* (2009 ▶). For synthetic details, see: Weber & Schleid (2000 ▶). For standard­ization of structural data, see: Gelato & Parthé (1987 ▶). For the VSEPR model, see: Gillespie (1970 ▶). For the bond-valence method, see: Brown (2009 ▶).

## Experimental

### 

#### Crystal data

La_10_Te_12_O_39_
*M* *_r_* = 3544.30
Triclinic, 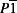

*a* = 5.6856 (11) Å
*b* = 12.621 (3) Å
*c* = 14.402 (3) Å
α = 95.53 (3)°
β = 100.88 (3)°
γ = 93.13 (3)°
*V* = 1007.3 (3) Å^3^
*Z* = 1
Mo *K*α radiation
μ = 18.98 mm^−1^
*T* = 293 K
0.12 × 0.04 × 0.02 mm

#### Data collection

Stoe IPDSII diffractometer
Absorption correction: numerical (*X-SHAPE* and *X-RED32*; Stoe, 2004 ▶) *T*_min_ = 0.256, *T*_max_ = 0.702
18743 measured reflections
8922 independent reflections
5110 reflections with *I* > 2σ(*I*)
*R*_int_ = 0.063

#### Refinement

*R*[*F* ^2^ > 2σ(*F* ^2^)] = 0.042
*wR*(*F* ^2^) = 0.064
*S* = 0.77
8922 reflections
289 parameters
Δρ_max_ = 3.71 e Å^−3^
Δρ_min_ = −3.98 e Å^−3^

### 

Data collection: *X-AREA* (Stoe, 2004 ▶); cell refinement: *X-AREA*; data reduction: *X-RED32* (Stoe, 2004 ▶); program(s) used to solve structure: *SHELXS97* (Sheldrick, 2008 ▶); program(s) used to refine structure: *SHELXL97* (Sheldrick, 2008 ▶); molecular graphics: *DIAMOND* (Crystal Impact, 2008 ▶); software used to prepare material for publication: *SHELXL97*.

## Supplementary Material

Crystal structure: contains datablock(s) global, I. DOI: 10.1107/S1600536813012191/wm2737sup1.cif

Structure factors: contains datablock(s) I. DOI: 10.1107/S1600536813012191/wm2737Isup2.hkl

Additional supplementary materials:  crystallographic information; 3D view; checkCIF report

## Figures and Tables

**Fig. 1.**
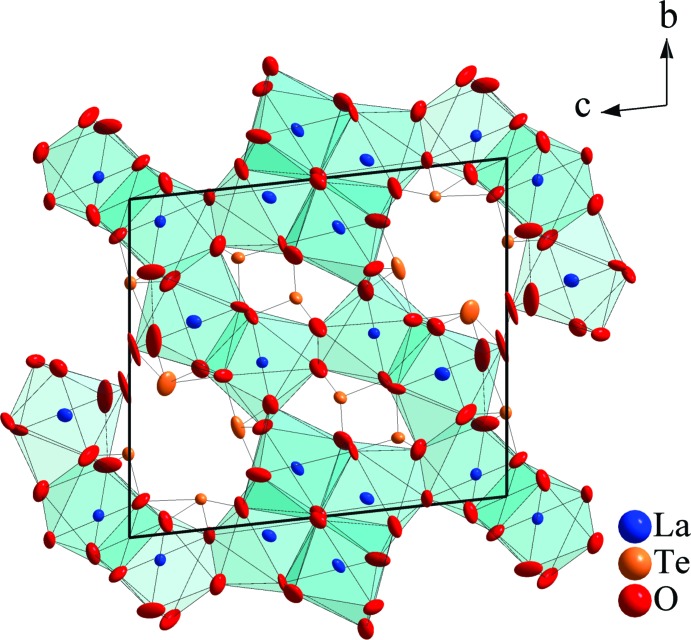
The crystal structure of La10Te12O39 represented with displacement ellipsoids at the 90% probability level.

## supplementary crystallographic information

### Comment

A number of rare-earth(III) oxotellurates(IV), including Dy_2_Te_3_O_9_ (Meier *et al.*, 2009), Nd_2_Te_4_O_11_ (Castro *et al.*, 1990) and Ho_2_Te_5_O_13_ (Weber *et al.*, 2001), have been prepared and structurally characterized. Despite their different compositions, these compounds all contain tellurium atoms in the oxidation state +IV. The remaining two electrons on the Te^IV^ atoms usually form stereo-chemically active but chemically non-bonding electron lone pairs. The local atomic arrangements around these Te^IV^ atoms with electron lone pairs can be understood on basis of the 'Valence Shell Electron Pair Repulsion' (VSEPR) model (Gillespie, 1970). As the local symmetry of the Te^IV^ sites is reduced by the lone pairs, these compounds usually adopt low-symmetry space groups, *e.g. C*2/*c* for Nd_2_Te_4_O_11_, *P*1 for Ho_2_Te_5_O_13_ or *P*2_1_/*c* for Dy_2_Te_3_O_9_. The title compound, La_10_Te_12_O_39_, can be considered as another member of the rare-earth(III) oxotellurates(IV) family. Structural similarities can be observed between La_10_Te_12_O_39_ and other rare-earth(III) oxotellurates(IV), including the distorted LaO_8_ polyhedra and a number of different Te–O structural motifs. Among the six Te atoms in the unit cell, four of them define empty channels parallel to the *a*-axis, while the other two fill the interstitial positions between the LaO_8_ polyhedra (Fig. 1).

If Te–O distances ≤ 2.03 Å (sum of the Te and O covalent radii) are considered as primary Te–O bonds, three TeO_3_ units (for Te1, Te4 and Te6) can be identified. Also, a fourth long Te–O interaction is observed for each of these three Te atoms (the fourth oxygen atoms is 2.852 (6) Å from Te1, 2.923 (8) Å from Te4 and 2.802 (6) Å from Te6). Bond valence sum calculations (Brown, 2009) show a minimal contribution from these long interactions; when these interactions are discarded, the bond valence sums for the 3-coordinated Te1, Te4 and Te6 atoms are close to 4.0 valence units (v.u.). The other three Te atoms (Te2, Te3 and Te5) display shorter secondary Te–O interactions (≤ 2.5 Å), which allow them to form TeO_4_ units in distorted seesaw configurations. The bond valence sums indicate significant contributions from these interactions. For these TeO_4_ units, bond valence sum are 3.9 v.u, 3.9 v.u and 4.4 v.u for Te2, Te3 and Te5, respectively. Such results are consistent with the assignment of oxidation state +IV for the Te atoms.

The competition between the Te2 and Te5 atoms to form stronger interactions with the bridging oxygen atom (O19) also causes disorder on the oxygen site. As a result, O19 is split into two separated oxygen positions (O191 and O192) to account for the electron density distribution. In addition to the disordered oxygen sites, one of the lanthanum sites (La5) is also described by split positions (La51 and La52). Such disorder could be a consequence of the asymmetric environment in the distorted LaO_8_ polyhedron.

### Experimental

Single crystals of La_10_Te_12_O_39_ were obtained by reacting La_2_O_3_ (99.99 wt. %, Rhône-Poulenc, pre-fired at 1273 K for 12 h) and TeO_2_ (99.99%, Johnson Matthey Electronics) in a CsCl (99.9%, Alfa-Aesar) flux (Weber & Schleid, 2000). In total, 0.1 gram of the La_2_O_3_ and TeO_2_ powder with the 1:2 molar ratio were ground and mixed with 1.9 grams of CsCl. The sample mixture was placed in an alumina crucible, which was sealed in a silica tube under vacuum. The sample was heated to 1273 K at a rate of 50 K/h. After holding at 1273 K for 72 h, the temperature was then slowly decreased to 1073 K at a rate of 5 K/hour. After annealing at 1073 K for 20 h, the sample was quenched in air. Transparent, colorless, needle-shaped single crystals were obtained by washing away the salt flux with distilled water and ethanol.

### Refinement

The structure was standardized by using the *STRUCTURE TIDY* program (Gelato & Parthé, 1987). Two pairs of split positions, La51/La52 and O191/O192 were modelled to account for the observed electron density distribution. Between each pair of spilt sites, the sum of their occupancies was constrained to 100%, while the isotropic or anisotropic displacement parameters were equalized using the EADP command. The occupancies of each site was extracted from the refinement. Deficiency on the O20 site was observed during the refinement. The occupancy of the O20 site was later assigned to 50% to account for charge balance. The remaining maximum and minimum electron densities (3.71 e^-^ Å^-3^ and -3.98 e^-^ Å^-3^) are 0.62 Å and 0.24 Å, respectively, from atom Te5.

### Crystal data

**Table:**

La_10_Te_12_O_39_	*Z* = 1
*M**_r_* = 3544.30	*F*(000) = 1506
Triclinic, *P*1	*D*_x_ = 5.843 Mg m^−^^3^
Hall symbol: -P 1	Mo *K*α radiation, λ = 0.71073 Å
*a* = 5.6856 (11) Å	Cell parameters from 18743 reflections
*b* = 12.621 (3) Å	θ = 2.9–36.9°
*c* = 14.402 (3) Å	µ = 18.98 mm^−^^1^
α = 95.53 (3)°	*T* = 293 K
β = 100.88 (3)°	Needle, colourless
γ = 93.13 (3)°	0.12 × 0.04 × 0.02 mm
*V* = 1007.3 (3) Å^3^	

### Data collection

**Table:**

Stoe IPDSII diffractometer	8922 independent reflections
Radiation source: fine-focus sealed tube	5110 reflections with *I* > 2σ(*I*)
Graphite monochromator	*R*_int_ = 0.063
ω–scan	θ_max_ = 36.3°, θ_min_ = 2.9°
Absorption correction: numerical (*X-SHAPE* and *X-RED32*; Stoe, 2004)	*h* = −9→6
*T*_min_ = 0.256, *T*_max_ = 0.702	*k* = −21→20
18743 measured reflections	*l* = −23→23

### Refinement

**Table:**

Refinement on *F*^2^	Primary atom site location: structure-invariant direct methods
Least-squares matrix: full	Secondary atom site location: difference Fourier map
*R*[*F*^2^ > 2σ(*F*^2^)] = 0.042	*w* = 1/[σ^2^(*F*_o_^2^) + (0.0122*P*)^2^] where *P* = (*F*_o_^2^ + 2*F*_c_^2^)/3
*wR*(*F*^2^) = 0.064	(Δ/σ)_max_ = 0.003
*S* = 0.77	Δρ_max_ = 3.71 e Å^−^^3^
8922 reflections	Δρ_min_ = −3.98 e Å^−^^3^
289 parameters	Extinction correction: *SHELXL97* (Sheldrick, 2008), Fc^*^=kFc[1+0.001xFc^2^λ^3^/sin(2θ)]^-1/4^
0 restraints	Extinction coefficient: 0.00195 (3)

### Special details

**Table:**

Experimental. A numerical absorption correction was based on the crystal shape that was originally derived from the optical face indexing but later optimized against equivalent reflections using Stoe *X-SHAPE* software (Stoe & Cie GmbH, 2004)
Geometry. All e.s.d.'s (except the e.s.d. in the dihedral angle between two l.s. planes) are estimated using the full covariance matrix. The cell e.s.d.'s are taken into account individually in the estimation of e.s.d.'s in distances, angles and torsion angles; correlations between e.s.d.'s in cell parameters are only used when they are defined by crystal symmetry. An approximate (isotropic) treatment of cell e.s.d.'s is used for estimating e.s.d.'s involving l.s. planes.
Refinement. Refinement of *F*^2^ against ALL reflections. The weighted *R*-factor *wR* and goodness of fit *S* are based on *F*^2^, conventional *R*-factors *R* are based on *F*, with *F* set to zero for negative *F*^2^. The threshold expression of *F*^2^ > σ(*F*^2^) is used only for calculating *R*-factors(gt) *etc*. and is not relevant to the choice of reflections for refinement. *R*-factors based on *F*^2^ are statistically about twice as large as those based on *F*, and *R*– factors based on ALL data will be even larger.

### Fractional atomic coordinates and isotropic or equivalent isotropic displacement parameters (Å^2^)

**Table:**

	*x*	*y*	*z*	*U*_iso_*/*U*_eq_	Occ. (<1)
La1	0.42088 (10)	0.52484 (4)	0.35191 (3)	0.00809 (10)	
La2	0.54271 (10)	0.15044 (4)	0.55453 (3)	0.01179 (12)	
La3	0.79356 (10)	0.07556 (4)	0.08168 (3)	0.00813 (9)	
La4	0.81948 (10)	0.38287 (4)	0.17100 (4)	0.01151 (11)	
La51	0.93544 (12)	0.03921 (6)	0.37049 (5)	0.01013 (15)	0.875 (2)
La52	0.9564 (9)	0.0092 (4)	0.3946 (4)	0.01013 (15)	0.125 (2)
Te1	0.03902 (11)	0.34576 (4)	0.44123 (4)	0.01010 (11)	
Te2	0.10966 (12)	0.29104 (6)	0.71241 (4)	0.01761 (14)	
Te3	0.22321 (11)	0.24798 (5)	0.00280 (4)	0.00909 (11)	
Te4	0.48089 (10)	0.20819 (4)	0.28804 (4)	0.01029 (11)	
Te5	0.33718 (14)	0.55670 (7)	0.09840 (5)	0.02424 (16)	
Te6	0.28887 (10)	0.90925 (4)	0.18953 (3)	0.00793 (10)	
O1	0.0464 (12)	0.8560 (5)	0.3423 (5)	0.0228 (15)	
O2	0.0751 (13)	0.2551 (6)	0.1077 (5)	0.0248 (15)	
O3	0.0812 (11)	0.3836 (5)	0.3226 (4)	0.0165 (13)	
O4	0.0853 (11)	0.1040 (5)	0.9697 (4)	0.0157 (13)	
O5	0.0957 (12)	0.0194 (5)	0.2117 (4)	0.0157 (13)	
O6	0.1285 (13)	0.6544 (6)	0.3738 (4)	0.0217 (14)	
O7	0.1599 (13)	0.5237 (5)	0.1880 (4)	0.0202 (14)	
O8	0.1863 (14)	0.2177 (6)	0.4339 (5)	0.0243 (15)	
O9	0.2484 (11)	0.0094 (5)	0.4996 (4)	0.0153 (13)	
O10	0.3008 (13)	0.4396 (6)	0.4992 (4)	0.0238 (16)	
O11	0.3612 (12)	0.2841 (5)	0.6470 (4)	0.0193 (13)	
O12	0.4366 (11)	0.9645 (5)	0.0960 (4)	0.0166 (12)	
O13	0.4386 (16)	0.4258 (8)	0.0647 (5)	0.039 (2)	
O14	0.4707 (11)	0.0531 (5)	0.7030 (4)	0.0195 (14)	
O15	0.5313 (11)	0.2219 (5)	0.0575 (5)	0.0253 (16)	
O16	0.5651 (11)	0.3536 (5)	0.2926 (4)	0.0139 (12)	
O17	0.6547 (11)	0.1833 (5)	0.4068 (4)	0.0139 (12)	
O18	0.7421 (12)	0.1666 (6)	0.2342 (4)	0.0207 (13)	
O191	0.290 (2)	0.4482 (9)	0.7515 (8)	0.0153 (18)	0.559 (13)
O192	0.382 (3)	0.4438 (11)	0.8010 (10)	0.0153 (18)	0.441 (13)
O20	0.916 (3)	0.4450 (15)	0.0126 (8)	0.032 (4)	0.50

### Atomic displacement parameters (Å^2^)

**Table:**

	*U*^11^	*U*^22^	*U*^33^	*U*^12^	*U*^13^	*U*^23^
La1	0.0086 (2)	0.0071 (2)	0.00811 (18)	0.00113 (17)	0.00047 (17)	0.00086 (15)
La2	0.0160 (3)	0.0123 (2)	0.0099 (2)	0.0065 (2)	0.0060 (2)	0.00511 (16)
La3	0.0085 (2)	0.00864 (19)	0.00750 (17)	0.00180 (17)	0.00207 (15)	0.00023 (14)
La4	0.0087 (2)	0.0109 (2)	0.0138 (2)	0.00191 (17)	−0.00131 (18)	0.00210 (17)
La51	0.0097 (2)	0.0098 (3)	0.0121 (3)	0.0027 (2)	0.0034 (2)	0.0037 (2)
La52	0.0097 (2)	0.0098 (3)	0.0121 (3)	0.0027 (2)	0.0034 (2)	0.0037 (2)
Te1	0.0108 (2)	0.0096 (2)	0.0100 (2)	−0.0011 (2)	0.00199 (18)	0.00261 (17)
Te2	0.0136 (3)	0.0306 (4)	0.0117 (2)	0.0098 (3)	0.0051 (2)	0.0082 (2)
Te3	0.0073 (2)	0.0111 (2)	0.00840 (19)	0.0012 (2)	0.00017 (18)	0.00111 (17)
Te4	0.0113 (2)	0.0080 (2)	0.0109 (2)	−0.00019 (19)	0.00045 (18)	0.00099 (16)
Te5	0.0210 (4)	0.0329 (4)	0.0213 (3)	0.0096 (3)	0.0112 (3)	−0.0031 (3)
Te6	0.0080 (2)	0.0077 (2)	0.00793 (19)	0.00001 (18)	0.00129 (18)	0.00082 (16)
O1	0.015 (3)	0.014 (3)	0.037 (4)	0.002 (2)	−0.004 (3)	0.008 (3)
O2	0.028 (4)	0.023 (4)	0.025 (3)	−0.007 (3)	0.019 (3)	−0.011 (3)
O3	0.016 (3)	0.019 (3)	0.012 (2)	−0.008 (2)	−0.003 (2)	0.008 (2)
O4	0.012 (3)	0.014 (3)	0.019 (3)	−0.003 (2)	0.002 (2)	−0.007 (2)
O5	0.022 (3)	0.017 (3)	0.008 (2)	0.010 (3)	0.001 (2)	0.001 (2)
O6	0.026 (4)	0.026 (3)	0.015 (3)	0.014 (3)	0.003 (3)	0.004 (2)
O7	0.022 (3)	0.020 (3)	0.019 (3)	0.005 (3)	0.000 (3)	0.006 (2)
O8	0.030 (4)	0.022 (3)	0.022 (3)	0.013 (3)	−0.001 (3)	0.009 (2)
O9	0.011 (3)	0.017 (3)	0.017 (3)	0.000 (2)	−0.003 (2)	0.007 (2)
O10	0.024 (3)	0.023 (3)	0.019 (3)	−0.013 (3)	−0.007 (3)	0.008 (2)
O11	0.022 (3)	0.013 (3)	0.024 (3)	0.001 (3)	0.011 (3)	−0.004 (2)
O12	0.011 (3)	0.029 (3)	0.012 (2)	0.003 (3)	0.004 (2)	0.007 (2)
O13	0.038 (5)	0.063 (6)	0.014 (3)	0.035 (4)	−0.005 (3)	0.001 (3)
O14	0.015 (3)	0.029 (3)	0.012 (2)	−0.008 (3)	−0.005 (2)	0.008 (2)
O15	0.009 (3)	0.014 (3)	0.046 (4)	0.003 (2)	−0.011 (3)	0.002 (3)
O16	0.014 (3)	0.008 (2)	0.020 (3)	0.003 (2)	0.001 (2)	0.005 (2)
O17	0.019 (3)	0.020 (3)	0.006 (2)	0.006 (2)	0.006 (2)	0.006 (2)
O18	0.022 (3)	0.025 (3)	0.015 (2)	0.006 (3)	0.005 (2)	−0.005 (2)
O191	0.018 (5)	0.011 (4)	0.021 (4)	0.004 (4)	0.012 (4)	0.002 (3)
O192	0.018 (5)	0.011 (4)	0.021 (4)	0.004 (4)	0.012 (4)	0.002 (3)
O20	0.028 (8)	0.066 (11)	0.010 (5)	0.026 (8)	0.007 (5)	0.020 (6)

### Geometric parameters (Å, º)

**Table:**

La1—O10^i^	2.400 (6)	Te2—O191	2.156 (11)
La1—O6	2.435 (7)	Te2—O192	2.488 (14)
La1—O191^i^	2.448 (12)	Te3—O15	1.841 (6)
La1—O16	2.485 (6)	Te3—O2	1.861 (7)
La1—O3	2.502 (6)	Te3—O4^ix^	1.919 (6)
La1—O7	2.539 (6)	Te3—O13	2.496 (10)
La1—O11^i^	2.647 (6)	Te4—O16	1.863 (6)
La1—O10	2.652 (7)	Te4—O17	1.871 (5)
La1—O192^i^	2.706 (15)	Te4—O18	1.877 (7)
La2—O9	2.345 (6)	Te5—O13	1.831 (9)
La2—O17	2.400 (6)	Te5—O7	1.845 (7)
La2—O11	2.441 (6)	Te5—O20^vi^	1.936 (13)
La2—O9^ii^	2.528 (7)	Te5—O192^i^	1.945 (15)
La2—O1^i^	2.531 (6)	Te6—O5^x^	1.859 (7)
La2—O8	2.643 (6)	Te6—O14^i^	1.866 (5)
La2—O14	2.651 (6)	Te6—O12	1.883 (6)
La2—O6^i^	2.985 (7)	O1—Te2^viii^	2.032 (7)
La3—O15	2.447 (6)	O1—La52^xi^	2.131 (9)
La3—O18	2.454 (6)	O1—La51^xi^	2.446 (7)
La3—O12^iii^	2.456 (6)	O1—La2^i^	2.531 (6)
La3—O5^iv^	2.477 (6)	O2—La4^xii^	2.453 (8)
La3—O4^ii^	2.489 (7)	O2—La3^xii^	2.655 (7)
La3—O4^v^	2.557 (7)	O3—La4^xii^	2.398 (5)
La3—O12^vi^	2.633 (5)	O4—Te3^xiii^	1.919 (6)
La3—O2^iv^	2.655 (7)	O4—La3^ii^	2.489 (7)
La4—O3^iv^	2.398 (5)	O4—La3^xiv^	2.557 (7)
La4—O2^iv^	2.453 (8)	O5—Te6^iii^	1.859 (7)
La4—O191^i^	2.476 (11)	O5—La3^xii^	2.477 (6)
La4—O16	2.513 (7)	O5—La51^xii^	2.612 (6)
La4—O7^iv^	2.516 (7)	O5—La52^xii^	2.902 (8)
La4—O13	2.522 (7)	O6—Te2^viii^	1.866 (6)
La4—O192^i^	2.556 (15)	O6—La2^i^	2.985 (7)
La4—O20	2.626 (13)	O7—La4^xii^	2.516 (7)
La4—O15	2.733 (6)	O8—La51^xii^	2.589 (7)
La4—O18	2.995 (7)	O8—La52^xii^	2.829 (9)
La51—O9^iv^	2.393 (6)	O9—La52^xii^	2.023 (7)
La51—O9^ii^	2.420 (7)	O9—La52^ii^	2.107 (9)
La51—O1^vii^	2.446 (7)	O9—La51^xii^	2.393 (6)
La51—O14^ii^	2.521 (6)	O9—La51^ii^	2.420 (7)
La51—O17	2.565 (7)	O9—La2^ii^	2.528 (7)
La51—O8^iv^	2.589 (7)	O10—La1^i^	2.400 (6)
La51—O5^iv^	2.612 (6)	O11—La1^i^	2.647 (6)
La51—O18	2.763 (7)	O12—La3^x^	2.456 (6)
La52—O9^iv^	2.023 (7)	O12—La3^vi^	2.633 (5)
La52—O9^ii^	2.107 (9)	O14—Te6^i^	1.866 (5)
La52—O1^vii^	2.131 (9)	O14—La51^ii^	2.521 (6)
La52—O14^ii^	2.604 (7)	O14—La52^ii^	2.604 (7)
La52—O8^iv^	2.829 (9)	O191—O192	0.808 (16)
La52—O17	2.872 (8)	O191—La1^i^	2.448 (12)
La52—O5^iv^	2.902 (8)	O191—La4^i^	2.476 (11)
Te1—O10	1.861 (6)	O192—Te5^i^	1.945 (15)
Te1—O8	1.864 (7)	O192—La4^i^	2.556 (15)
Te1—O3	1.872 (6)	O192—La1^i^	2.706 (15)
Te2—O11	1.857 (7)	O20—O20^xv^	1.75 (4)
Te2—O6^viii^	1.866 (6)	O20—Te5^vi^	1.936 (13)
Te2—O1^viii^	2.032 (7)		
			
O10^i^—La1—O6	100.8 (2)	O9^ii^—La51—O5^iv^	159.6 (2)
O10^i^—La1—O191^i^	97.2 (3)	O1^vii^—La51—O5^iv^	71.5 (2)
O6—La1—O191^i^	121.3 (3)	O14^ii^—La51—O5^iv^	94.0 (2)
O10^i^—La1—O16	99.0 (2)	O17—La51—O5^iv^	122.32 (18)
O6—La1—O16	156.5 (2)	O8^iv^—La51—O5^iv^	94.0 (2)
O191^i^—La1—O16	68.0 (3)	O9^iv^—La51—O18	149.6 (2)
O10^i^—La1—O3	123.0 (2)	O9^ii^—La51—O18	125.3 (2)
O6—La1—O3	87.4 (2)	O1^vii^—La51—O18	126.6 (2)
O191^i^—La1—O3	126.1 (3)	O14^ii^—La51—O18	75.5 (2)
O16—La1—O3	71.2 (2)	O17—La51—O18	58.70 (18)
O10^i^—La1—O7	168.4 (2)	O8^iv^—La51—O18	79.75 (19)
O6—La1—O7	75.5 (2)	O5^iv^—La51—O18	64.02 (19)
O191^i^—La1—O7	76.1 (3)	O9^iv^—La52—O9^ii^	86.2 (3)
O16—La1—O7	87.4 (2)	O9^iv^—La52—O1^vii^	86.6 (3)
O3—La1—O7	68.2 (2)	O9^ii^—La52—O1^vii^	108.8 (4)
O10^i^—La1—O11^i^	73.2 (2)	O9^iv^—La52—O14^ii^	156.7 (4)
O6—La1—O11^i^	73.3 (2)	O9^ii^—La52—O14^ii^	76.7 (3)
O191^i^—La1—O11^i^	59.6 (3)	O1^vii^—La52—O14^ii^	84.1 (3)
O16—La1—O11^i^	125.0 (2)	O9^iv^—La52—O8^iv^	71.1 (3)
O3—La1—O11^i^	157.5 (2)	O9^ii^—La52—O8^iv^	106.8 (3)
O7—La1—O11^i^	95.2 (2)	O1^vii^—La52—O8^iv^	136.2 (3)
O10^i^—La1—O10	63.2 (3)	O14^ii^—La52—O8^iv^	128.9 (3)
O6—La1—O10	86.3 (2)	O9^iv^—La52—O17	117.3 (3)
O191^i^—La1—O10	149.8 (3)	O9^ii^—La52—O17	69.9 (3)
O16—La1—O10	91.4 (2)	O1^vii^—La52—O17	155.5 (3)
O3—La1—O10	61.16 (18)	O14^ii^—La52—O17	71.7 (2)
O7—La1—O10	126.7 (2)	O8^iv^—La52—O17	62.9 (2)
O11^i^—La1—O10	127.04 (19)	O9^iv^—La52—O5^iv^	110.9 (3)
O10^i^—La1—O192^i^	113.7 (4)	O9^ii^—La52—O5^iv^	162.5 (3)
O6—La1—O192^i^	111.4 (4)	O1^vii^—La52—O5^iv^	70.1 (3)
O191^i^—La1—O192^i^	17.1 (4)	O14^ii^—La52—O5^iv^	85.8 (2)
O16—La1—O192^i^	71.3 (3)	O8^iv^—La52—O5^iv^	83.1 (2)
O3—La1—O192^i^	115.1 (3)	O17—La52—O5^iv^	103.5 (2)
O7—La1—O192^i^	59.1 (4)	O10—Te1—O8	100.6 (3)
O11^i^—La1—O192^i^	63.8 (3)	O10—Te1—O3	89.4 (3)
O10—La1—O192^i^	162.1 (4)	O8—Te1—O3	96.2 (3)
O10^i^—La1—Te1	93.63 (16)	O11—Te2—O6^viii^	102.3 (3)
O9—La2—O17	99.5 (2)	O11—Te2—O1^viii^	95.2 (3)
O9—La2—O11	106.8 (2)	O6^viii^—Te2—O1^viii^	86.7 (3)
O17—La2—O11	122.0 (2)	O11—Te2—O191	78.0 (4)
O9—La2—O9^ii^	72.4 (3)	O6^viii^—Te2—O191	90.4 (4)
O17—La2—O9^ii^	72.6 (2)	O1^viii^—Te2—O191	171.9 (4)
O11—La2—O9^ii^	164.7 (2)	O11—Te2—O192	79.9 (4)
O9—La2—O1^i^	128.0 (2)	O6^viii^—Te2—O192	108.0 (4)
O17—La2—O1^i^	100.2 (2)	O1^viii^—Te2—O192	165.2 (4)
O11—La2—O1^i^	102.3 (2)	O191—Te2—O192	18.3 (4)
O9^ii^—La2—O1^i^	68.6 (2)	O15—Te3—O2	101.3 (3)
O9—La2—O8	70.7 (2)	O15—Te3—O4^ix^	99.7 (3)
O17—La2—O8	67.6 (2)	O2—Te3—O4^ix^	88.2 (3)
O11—La2—O8	73.9 (2)	O15—Te3—O13	73.6 (3)
O9^ii^—La2—O8	118.8 (2)	O2—Te3—O13	89.1 (3)
O1^i^—La2—O8	160.4 (2)	O4^ix^—Te3—O13	172.1 (2)
O9—La2—O14	72.04 (19)	O16—Te4—O17	99.0 (3)
O17—La2—O14	161.3 (2)	O16—Te4—O18	94.0 (3)
O11—La2—O14	76.7 (2)	O17—Te4—O18	88.6 (3)
O9^ii^—La2—O14	88.8 (2)	O13—Te5—O7	101.0 (4)
O1^i^—La2—O14	74.1 (2)	O13—Te5—O20^vi^	95.8 (6)
O8—La2—O14	122.3 (2)	O7—Te5—O20^vi^	100.5 (5)
O9—La2—O6^i^	173.5 (2)	O13—Te5—O192^i^	80.4 (5)
O17—La2—O6^i^	82.1 (2)	O7—Te5—O192^i^	86.2 (5)
O11—La2—O6^i^	67.3 (2)	O20^vi^—Te5—O192^i^	172.9 (6)
O9^ii^—La2—O6^i^	114.1 (2)	O5^x^—Te6—O14^i^	96.7 (3)
O1^i^—La2—O6^i^	57.4 (2)	O5^x^—Te6—O12	99.5 (3)
O8—La2—O6^i^	104.5 (2)	O14^i^—Te6—O12	100.2 (3)
O14—La2—O6^i^	108.17 (17)	Te2^viii^—O1—La52^xi^	140.8 (4)
O15—La3—O18	68.9 (2)	Te2^viii^—O1—La51^xi^	139.5 (3)
O15—La3—O12^iii^	87.2 (2)	La52^xi^—O1—La51^xi^	11.06 (15)
O18—La3—O12^iii^	83.6 (2)	Te2^viii^—O1—La2^i^	113.0 (3)
O15—La3—O5^iv^	137.5 (2)	La52^xi^—O1—La2^i^	100.1 (3)
O18—La3—O5^iv^	70.7 (2)	La51^xi^—O1—La2^i^	105.8 (2)
O12^iii^—La3—O5^iv^	100.8 (2)	Te3—O2—La4^xii^	133.6 (4)
O15—La3—O4^ii^	150.80 (19)	Te3—O2—La3^xii^	104.2 (3)
O18—La3—O4^ii^	134.4 (2)	La4^xii^—O2—La3^xii^	101.3 (3)
O12^iii^—La3—O4^ii^	80.0 (2)	Te1—O3—La4^xii^	134.1 (3)
O5^iv^—La3—O4^ii^	71.1 (2)	Te1—O3—La1	107.3 (2)
O15—La3—O4^v^	103.2 (2)	La4^xii^—O3—La1	113.3 (2)
O18—La3—O4^v^	134.4 (2)	Te3^xiii^—O4—La3^ii^	139.3 (3)
O12^iii^—La3—O4^v^	141.94 (19)	Te3^xiii^—O4—La3^xiv^	106.1 (3)
O5^iv^—La3—O4^v^	95.7 (2)	La3^ii^—O4—La3^xiv^	106.7 (2)
O4^ii^—La3—O4^v^	73.3 (2)	Te6^iii^—O5—La3^xii^	121.3 (3)
O15—La3—O12^vi^	75.3 (2)	Te6^iii^—O5—La51^xii^	119.3 (3)
O18—La3—O12^vi^	140.1 (2)	La3^xii^—O5—La51^xii^	109.6 (3)
O12^iii^—La3—O12^vi^	77.4 (2)	Te6^iii^—O5—La52^xii^	109.8 (3)
O5^iv^—La3—O12^vi^	147.1 (2)	La3^xii^—O5—La52^xii^	117.1 (3)
O4^ii^—La3—O12^vi^	76.3 (2)	La51^xii^—O5—La52^xii^	9.51 (11)
O4^v^—La3—O12^vi^	70.38 (19)	Te2^viii^—O6—La1	131.9 (3)
O15—La3—O2^iv^	72.9 (2)	Te2^viii^—O6—La2^i^	101.1 (3)
O18—La3—O2^iv^	74.6 (2)	La1—O6—La2^i^	100.1 (2)
O12^iii^—La3—O2^iv^	154.5 (2)	Te5—O7—La4^xii^	128.2 (3)
O5^iv^—La3—O2^iv^	84.6 (2)	Te5—O7—La1	112.1 (3)
O4^ii^—La3—O2^iv^	124.9 (2)	La4^xii^—O7—La1	108.2 (2)
O4^v^—La3—O2^iv^	60.6 (2)	Te1—O8—La51^xii^	120.8 (3)
O12^vi^—La3—O2^iv^	111.4 (2)	Te1—O8—La2	129.5 (3)
O15—La3—Te3^iv^	84.65 (18)	La51^xii^—O8—La2	100.7 (2)
O18—La3—Te3^iv^	104.67 (17)	Te1—O8—La52^xii^	127.0 (4)
O12^iii^—La3—Te3^iv^	165.27 (13)	La51^xii^—O8—La52^xii^	10.27 (10)
O5^iv^—La3—Te3^iv^	93.53 (16)	La2—O8—La52^xii^	91.0 (2)
O4^ii^—La3—Te3^iv^	101.55 (16)	La52^xii^—O9—La52^ii^	93.8 (3)
O4^v^—La3—Te3^iv^	30.83 (13)	La52^xii^—O9—La2	126.6 (3)
O12^vi^—La3—Te3^iv^	88.70 (14)	La52^ii^—O9—La2	108.6 (3)
O2^iv^—La3—Te3^iv^	30.10 (14)	La52^xii^—O9—La51^xii^	10.31 (16)
O3^iv^—La4—O2^iv^	87.2 (2)	La52^ii^—O9—La51^xii^	99.6 (3)
O3^iv^—La4—O191^i^	81.8 (3)	La2—O9—La51^xii^	116.4 (3)
O2^iv^—La4—O191^i^	158.2 (3)	La52^xii^—O9—La51^ii^	101.2 (3)
O3^iv^—La4—O16	71.8 (2)	La52^ii^—O9—La51^ii^	11.21 (15)
O2^iv^—La4—O16	126.8 (2)	La2—O9—La51^ii^	109.7 (2)
O191^i^—La4—O16	67.2 (3)	La51^xii^—O9—La51^ii^	108.1 (3)
O3^iv^—La4—O7^iv^	70.2 (2)	La52^xii^—O9—La2^ii^	103.2 (3)
O2^iv^—La4—O7^iv^	88.0 (2)	La52^ii^—O9—La2^ii^	117.3 (3)
O191^i^—La4—O7^iv^	70.6 (3)	La2—O9—La2^ii^	107.6 (3)
O16—La4—O7^iv^	125.9 (2)	La51^xii^—O9—La2^ii^	107.5 (2)
O3^iv^—La4—O13	153.1 (3)	La51^ii^—O9—La2^ii^	107.1 (2)
O2^iv^—La4—O13	119.0 (2)	Te1—O10—La1^i^	139.7 (3)
O191^i^—La4—O13	75.2 (3)	Te1—O10—La1	102.0 (2)
O16—La4—O13	86.4 (3)	La1^i^—O10—La1	116.8 (3)
O7^iv^—La4—O13	113.6 (3)	Te2—O11—La2	138.5 (3)
O3^iv^—La4—O192^i^	100.2 (4)	Te2—O11—La1^i^	111.5 (3)
O2^iv^—La4—O192^i^	159.8 (4)	La2—O11—La1^i^	110.0 (3)
O191^i^—La4—O192^i^	18.4 (4)	Te6—O12—La3^x^	140.4 (3)
O16—La4—O192^i^	73.4 (4)	Te6—O12—La3^vi^	117.0 (3)
O7^iv^—La4—O192^i^	77.0 (4)	La3^x^—O12—La3^vi^	102.6 (2)
O13—La4—O192^i^	57.4 (4)	Te5—O13—Te3	133.3 (4)
O3^iv^—La4—O20	129.1 (4)	Te5—O13—La4	113.8 (4)
O2^iv^—La4—O20	71.6 (4)	Te3—O13—La4	104.4 (3)
O191^i^—La4—O20	101.1 (5)	Te6^i^—O14—La51^ii^	149.0 (3)
O16—La4—O20	155.9 (3)	Te6^i^—O14—La52^ii^	157.7 (4)
O7^iv^—La4—O20	63.5 (4)	La51^ii^—O14—La52^ii^	11.98 (12)
O13—La4—O20	70.0 (4)	Te6^i^—O14—La2	112.9 (3)
O192^i^—La4—O20	89.4 (5)	La51^ii^—O14—La2	97.73 (17)
O3^iv^—La4—O15	131.0 (2)	La52^ii^—O14—La2	87.0 (2)
O2^iv^—La4—O15	71.4 (2)	Te3—O15—La3	141.2 (3)
O191^i^—La4—O15	129.5 (3)	Te3—O15—La4	119.0 (3)
O16—La4—O15	86.3 (2)	La3—O15—La4	99.34 (19)
O7^iv^—La4—O15	147.7 (2)	Te4—O16—La1	137.7 (3)
O13—La4—O15	60.4 (3)	Te4—O16—La4	109.3 (3)
O192^i^—La4—O15	115.1 (4)	La1—O16—La4	110.7 (2)
O20—La4—O15	85.9 (4)	Te4—O17—La2	133.5 (3)
O3^iv^—La4—O18	73.59 (18)	Te4—O17—La51	105.0 (2)
O2^iv^—La4—O18	68.6 (2)	La2—O17—La51	106.5 (2)
O191^i^—La4—O18	125.1 (3)	Te4—O17—La52	112.6 (3)
O16—La4—O18	58.79 (19)	La2—O17—La52	97.1 (2)
O7^iv^—La4—O18	137.49 (19)	La51—O17—La52	9.40 (11)
O13—La4—O18	108.8 (3)	Te4—O18—La3	135.8 (3)
O192^i^—La4—O18	131.5 (4)	Te4—O18—La51	97.8 (3)
O20—La4—O18	132.5 (4)	La3—O18—La51	105.6 (2)
O15—La4—O18	57.66 (18)	Te4—O18—La4	92.0 (3)
O9^iv^—La51—O9^ii^	71.9 (3)	La3—O18—La4	92.5 (2)
La52—La51—O1^vii^	49.1 (5)	La51—O18—La4	141.2 (2)
O9^iv^—La51—O1^vii^	72.2 (2)	O192—O191—Te2	104.8 (14)
O9^ii^—La51—O1^vii^	90.2 (2)	O192—O191—La1^i^	99.9 (15)
La52—La51—O14^ii^	92.8 (5)	Te2—O191—La1^i^	108.9 (5)
O9^iv^—La51—O14^ii^	134.6 (2)	O192—O191—La4^i^	86.4 (13)
O9^ii^—La51—O14^ii^	73.25 (19)	Te2—O191—La4^i^	133.7 (6)
O1^vii^—La51—O14^ii^	79.9 (2)	La1^i^—O191—La4^i^	113.2 (4)
La52—La51—O17	119.8 (6)	O191—O192—Te5^i^	165.3 (18)
O9^iv^—La51—O17	115.9 (2)	O191—O192—Te2	56.9 (12)
O9^ii^—La51—O17	71.7 (2)	Te5^i^—O192—Te2	129.3 (7)
O1^vii^—La51—O17	155.0 (2)	O191—O192—La4^i^	75.2 (13)
O14^ii^—La51—O17	78.4 (2)	Te5^i^—O192—La4^i^	108.3 (6)
La52—La51—O8^iv^	111.1 (5)	Te2—O192—La4^i^	115.3 (6)
O9^iv^—La51—O8^iv^	71.0 (2)	O191—O192—La1^i^	63.0 (14)
O9^ii^—La51—O8^iv^	105.4 (2)	Te5^i^—O192—La1^i^	102.4 (6)
O1^vii^—La51—O8^iv^	132.6 (2)	Te2—O192—La1^i^	92.3 (5)
O14^ii^—La51—O8^iv^	147.3 (2)	La4^i^—O192—La1^i^	102.7 (5)
O17—La51—O8^iv^	70.5 (2)	O20^xv^—O20—Te5^vi^	95.3 (9)
La52—La51—O5^iv^	117.6 (5)	O20^xv^—O20—La4	133.2 (12)
O9^iv^—La51—O5^iv^	109.5 (2)	Te5^vi^—O20—La4	120.3 (8)

Symmetry codes: (i) −*x*+1, −*y*+1, −*z*+1; (ii) −*x*+1, −*y*, −*z*+1; (iii) *x*, *y*−1, *z*; (iv) *x*+1, *y*, *z*; (v) *x*+1, *y*, *z*−1; (vi) −*x*+1, −*y*+1, −*z*; (vii) *x*+1, *y*−1, *z*; (viii) −*x*, −*y*+1, −*z*+1; (ix) *x*, *y*, *z*−1; (x) *x*, *y*+1, *z*; (xi) *x*−1, *y*+1, *z*; (xii) *x*−1, *y*, *z*; (xiii) *x*, *y*, *z*+1; (xiv) *x*−1, *y*, *z*+1; (xv) −*x*+2, −*y*+1, −*z*.
